# Social Media–Based Interventions for Health Behavior Change in Low- and Middle-Income Countries: Systematic Review

**DOI:** 10.2196/31889

**Published:** 2022-04-14

**Authors:** Jessie Seiler, Tanya E Libby, Emahlea Jackson, JR Lingappa, WD Evans

**Affiliations:** 1 Department of Epidemiology University of Washington Seattle, WA United States; 2 Departments of Global Health, Medicine, and Pediatrics University of Washington Seattle, WA United States; 3 Departments of Prevention and Community Health & Global Health George Washington University Washington, DC United States

**Keywords:** social media, behavior change, low- and middle-income countries, mobile phone

## Abstract

**Background:**

Despite the wealth of evidence regarding effective health behavior change techniques using digital interventions to focus on residents of high-income countries, there is limited information of a similar nature for low- and middle-income countries.

**Objective:**

The aim of this review is to identify and describe the available literature on effective social media–based behavior change interventions within low- and middle-income countries.

**Methods:**

This systematic review was conducted in accordance with the 2009 PRISMA (Preferred Reporting Items for Systematic Reviews and Meta-Analyses) guidelines. We searched PubMed, Embase, Elsevier, CINAHL, PsycInfo, and Global Index Medicus, and the final search was conducted on April 6, 2021. We excluded studies published before 2000 because of the subject matter. We included studies that evaluated interventions conducted at least partly on a social media platform.

**Results:**

We identified 1832 studies, of which 108 (5.89%) passed title-abstract review and were evaluated by full-text review. In all, 30.6% (33/108) were included in the final analysis. Although 22 studies concluded that the social media intervention was effective, only 13 quantified the level of social media engagement, of which, few used theory (n=8) or a conceptual model (n=5) of behavior change.

**Conclusions:**

We identified gaps in the settings of interventions, types and sectors of interventions, length of follow-up, evaluation techniques, use of theoretical and conceptual models, and discussions of the privacy implications of social media use.

**Trial Registration:**

PROSPERO CRD42020223572; https://www.crd.york.ac.uk/prospero/display_record.php?RecordID=223572

## Introduction

### Background

Social media platforms are ubiquitous in modern world. Social media has become a widely used platform for advertisers, news media, and government agencies to reach billions of users as a form of connection and a means of communication with relatives, friends, businesses, colleagues, media figures, and acquaintances. For example, Meta (formerly known as Facebook) is a major conglomerate of social media networks, and it is estimated that 2.7 billion active users engaged with their flagship Facebook social media service in mid-2020 [1]. With such a wide reach and worldwide omnipresence, social media platforms—including Instagram, Twitter, Reddit, WeChat, and others—have been of increasing interest in the implementation of behavior change interventions and public health campaigns.

In a 2018 systematic review, Elaheebocus et al [2] determined that among peer-based social media interventions focused on tobacco smoking, nutrition, physical activity, or alcohol consumption, those with a sharing-enabled feature were most likely to elicit positive intervention outcomes. Results from multiple systematic reviews suggest that among adolescents and children, social media interventions—in comparison with in-person interventions—are underused; however, they may be effective tools for health promotion and behavior change [3-6].

Despite their potential utility, social media platforms have a checkered history of privacy and data theft. Of note is the mismatch in the intention versus actualization of privacy behavior and data sharing on social media platforms [7,8]. Although many people cite the desire to protect their privacy and limit the amount of information gathered by said platforms, average social media users do not exercise caution when granting access to third-party software or websites to use their data [7]. Given the diminished barrier to data collection as well as the high financial value and ease of access of said data, researchers have called for increased protection against malicious data exploits (eg, theft) and the development of ethical frameworks that encourage cautious behavior in both data collection and social media use [9-11].

Behavior change strategies implemented using digital technology include training, coaching, and text messages, all of which are potentially more effective with increased frequency, intensity, and follow-up [12]. Overall, the most effective health behavior change interventions use a combination of both digital and face-to-face components, lending credence to the importance of classical social behavior change modalities, including human interaction and in-person accountability [5,13-15]. The most commonly cited research gaps include multiple, noncomparable measures (eg, engagement and reach) to evaluate digital media–related behavior change campaigns [5,16,17]. Other areas highlighted for improvement include clarification of dose, intensity of intervention delivery, and measurement of long-term outcomes [17,18].

The preponderance of evidence characterizing effective behavior change techniques using digital interventions has been collected by focusing on residents of high-income countries. There are limited data of a similar nature for low- and middle-income countries (LMICs). A recent Pew Research study explored internet and smartphone use in LMICs and found a median of 67% of respondents reported having access to and using the internet, with 42% reporting access to smartphones in 2017 [19]. In the context of technology-based interventions for HIV prevention and care delivery, Maloney et al [20] found that, compared with high-income countries, the distinguishing characteristic of a successful intervention in LMICs appeared to be related to how well the intervention was tailored to serve the unique needs of a given community, village, or region, which was highly dependent on the culture within that group. Given the demonstrated need for these interventions to be crafted specifically for the setting of interest and the growing availability of technology in LMICs, a focus on behavior change interventions delivered over social media in LMICs is justified.

### Objectives

The goal of this review is to identify and describe the available literature regarding effective social media–based health behavior change interventions within LMICs.

## Methods

### Overview

We conducted a systematic review in concordance with the 2009 PRISMA (Preferred Reporting Items for Systematic Reviews and Meta-Analyses) guidelines to understand what behavioral interventions have been implemented using social media in LMICs and to characterize the evidence of their effectiveness [21]. One of our goals was to gather data on where and how these interventions are being implemented, including what subject areas have used social media interventions and for which outcomes. We also aimed to assess evaluation patterns, both in terms of the type of evaluations being carried out and whether social media interventions were deemed effective. Finally, we intended to collect information on funding sources, cost-effectiveness, and the use of theoretical and conceptual models. The review was registered with PROSPERO, where the review protocol can be viewed (registration number CRD42020223572).

### Search Strategy and Selection Criteria

We searched PubMed, Embase, Elsevier, CINAHL, PsycInfo, and Global Index Medicus for studies that detailed behavior change interventions with some component conducted on a social media platform. Our search terms, which were developed with the help of both subject matter experts and a librarian with expertise in conducting systematic reviews, encompassed both social media and health behavior change concepts and included terms to describe LMICs, as well as the names of all countries categorized as LMICs by the World Bank (Multimedia Appendix 1 provides full search threads). The final search for publications included in this review was conducted on April 6, 2021.

In recognition of the fact that social media is a relatively new format, we used search string limitations to exclude studies published before the year 2000. We also excluded studies for which we could not find a full-text version in English, including conference abstracts.

Studies were selected for review if they presented original evaluation data (formative, process or implementation, outcome or effectiveness, or impact related) for a behavior change intervention that was at least partly conducted over a social media platform and used the social components of the medium [22]. We included studies that examined changes in both behavior and health knowledge.

We excluded studies that did not describe a purposeful, planned intervention; accidental changes in service delivery that resulted in natural experiments were not of interest. We also excluded studies that included only 1-way communication, such as reminder text messages, as opposed to a multidirectional exchange of information, ideas, or opinions. For example, studies that described interventions that involved automated daily reminders for a certain behavior were excluded, as were studies that involved promulgating advertisements on a social media platform without evaluating engagement.

For our purposes, we defined social media relatively broadly and included search terms that would identify studies that used specific platforms that connect a network of individuals together for behavior change purposes, including both those tailor-made for the purposes of the intervention as well as the more established social networks that exist for commercial purposes (ie, Twitter, Facebook, and WhatsApp). However, we excluded studies that used social media to implement one-to-one conversations, such as conversations between health care providers and patients, as these conversations did not use the networking component of the apps.

In all, 2 independent reviewers used Covidence (Covidence systematic review software, Veritas Health Innovation) to screen each title and abstract to identify potentially eligible records (JS, TEL, or EJ). During screening, disagreements among the reviewers were settled through team consensus. If the disagreement could not be settled with information available in the title and abstract, the study was passed on to a full-text review. Full-text versions of potentially eligible records were reviewed and data were independently extracted by 2 reviewers, with discrepancies resolved through discussion and consensus (JS, TEL, or EJ).

### Data Extraction

We extracted information on each identified program, including its setting, intended audience, and intended behavior change; the method of measuring both exposure and outcome; the strength of the descriptions of the intervention and its social media components; the social media platform used and its role; observed outcomes; how they were evaluated; cost information; and whether their design and implementation were guided by the use of a theoretical framework or conceptual model. We also looked for information on how social media’s role in each intervention was described and evaluated. Data extraction was conducted by a single reviewer for each study, with 10% of the manuscripts and extracted data selected randomly for quality control checking by a second reviewer (JS, TEL, or EJ).

The evaluation-specific information extracted for each study varied depending on the stage of evaluation assessed in the publication. For the process or implementation evaluations, we looked for information on the focus of the interventions and barriers to and facilitators of implementation. For outcome evaluations, we looked for the same indicators as we did for process or implementation evaluations along with data on changed behaviors. For impact evaluations, we collected all the indicators already mentioned as well as data on changes in health outcomes among participants.

For all studies, we searched for information on funding and cost-effectiveness associated with the program. We also extracted information on whether and how each intervention used a theoretical framework in its design and implementation.

### Risk of Bias Assessment

For each study, we conducted a qualitative assessment of the potential for bias based on the available descriptions of methods for inclusion in the intervention and analysis of the results, when available. We also noted the potential for bias, as recorded in the *Limitations* section.

## Results

### General Characteristics of Included Studies

**Figure 1:**
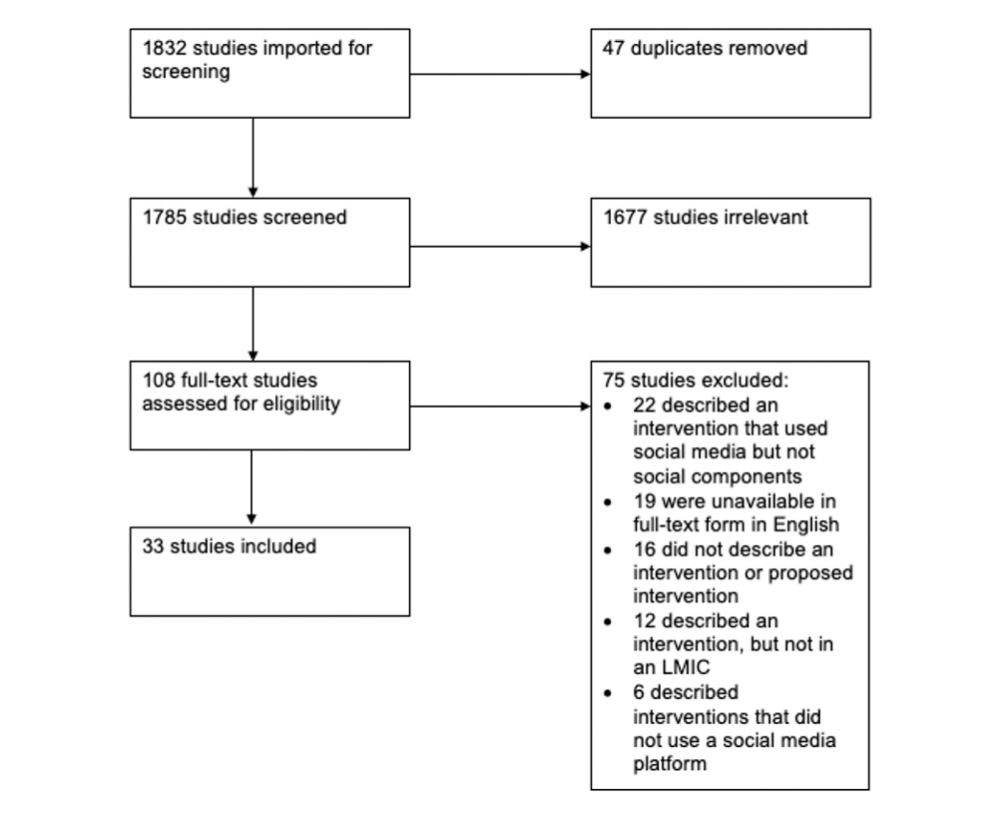
PRISMA (Preferred Reporting Items for Systematic Reviews and Meta-Analyses) flow diagram. LMIC: low- and middle-income country.

A total of 1832 studies were identified based on the search strategy, of which 108 (5.89%) passed title-abstract review and were evaluated by full-text review (Figure 1). At full-text review, 75 studies were excluded: 22 (29%) for using a social media platform without using the networking capabilities of these platforms (ie, for using only one-to-one communications), 19 (25%) because no version of the full text of the study could be found in English, 16 (21%) because no intervention was described, 12 (16%) because the intervention did not take place in an LMIC, and 6 (8%) because the intervention did not use a social media platform at all. Finally, 33 studies were included in this review [23-55]. The key study characteristics are summarized in Tables 1 and 2.

**Table 1:** Characteristics of the included studies.

Study	Setting	Target audience of behavior	Desired behavior change	Social media platform used	Length of follow-up
Garett et al [23]	Lima metro area, Peru	MSM^a^ adults at high risk of HIV	HIV testing	Facebook	1 year
Young et al [24]	Lima metro area, Peru	MSM adults at high risk of HIV	HIV testing	Facebook	1 year
Harding et al [25]	Ghana	People who breastfeed or might be in a position to support or promote breastfeeding	Breastfeeding and supporting breastfeeding	Facebook and Twitter	No information available
Cao et al [26]	Guangdong and Shandong provinces, China	MSM aged >16 years	HIV testing	WhatsApp	3 months
Sap et al [27]	Cameroon	Adolescents and young adults living with diabetes	Diabetes knowledge and glycemic control	Not specified	2 months
Cole et al [28]	Kerala, India	People living in the area of a Nipah virus outbreak	Nipah virus knowledge	Reddit	No information available
Hutchinson et al [29]	Kenya	Adolescents and young adults	Increased knowledge and changed behaviors around family planning and income generation	Facebook, Twitter, Instagram, YouTube, and WhatsApp	1 year
Goldenhersch et al [30]	Buenos Aires, Argentina	Smokers aged between 26 and 65 years	Tobacco cessation	Created for the intervention	90 days
Cool et al [31]	The Philippines	General population	Emergency response to typhoon	Facebook, Twitter, and Instagram	No information available
Hamill et al [32]	Alexandria, Egypt	General population	Build awareness and support for an upcoming smoking ban	Facebook	1 month
Jiebing et al [33]	Tibet	Pregnant women	Increase number of prenatal care visits	WeChat	Length of pregnancy
Mo et al [34]	Shanghai, China	Undergraduate students	Increased physical activity	WeChat	7 weeks
Wu et al [35]	Guangzhou and Shenzhen, China	MSM	Increase STI^b^ testing and other health-seeking activities	Created for the intervention	No information available
Ahmad et al [36]	Selangor, Malaysia	Parents and their primary school-going children (aged 8-11 years) who were overweight or obese	Physical activity, healthy diet, and reduced screen time	WhatsApp and Facebook	6 months
Cavalcanti et al [37]	João Pessoa city, Brazil	Mothers who recently gave birth	Breastfeeding	Facebook	6 months
Chen et al [38]	China	Chinese men aged 25-44 years who smoke tobacco	Smoking cessation	WeChat	6 weeks
Todorovic et al [39]	Belgrade, Serbia	First and fifth-year medical students	Physical activity	Facebook	1 month
Chai et al [40]	Zhongshan City, Guangdong Province, China	Employees of labor-intensive manufacturing factories aged >16 years	Smoking cessation	WeChat	1 month
Lwin et al [41]	Sri Lanka	General population	Dengue knowledge and prevention strategies	Created for the intervention	No information available
Pereira et al [42]	Brazil	General population	Uptake of HPV^c^ vaccine	Facebook	No information available
Gamboa et al [43]	La Vega, San Francisco, and Puerto Plata; Dominican Republic	Youth aged 14-18 years in communities at high risk for arboviruses	Zika prevention behaviors	Facebook	No information available
Januraga et al [44]	Indonesia	Urban, unmarried adolescent girls aged 16-19 years	Healthy diet	Facebook, Instagram, YouTube, and another app created for the intervention	No information available
Thammasarn and Banchonhattakit [45]	Nakhon Ratchasima Province, Thailand	Senior-primary school students	Physical activity and healthy diet	Not specified	No information available
He et al [46]	China	General population	Weight loss	WeChat	No information available
Souza et al [47]	Brazil	Community leaders	Civic engagement in general public health work	WhatsApp	No information available
Chiu et al [48]	Peru	MSM	HIV prevention and testing	Facebook	No information available
Chiu et al [49]	Peru	MSM	HIV prevention and testing	Facebook	No information available
Purdy [50]	Turkey	General population	Condom use	Facebook	No information available
Parsapure et al [51]	Kermanshah, Iran	General population	Vaginal health	Not specified	6 months
Wu et al [52]	Huzhu County, Qinghai Province, China	Breastfeeding mothers aged >17 years	Breastfeeding	WeChat	5 months
Hutchinson et al [53]	Ghana	Adolescent girls	Refusal to smoke tobacco	Facebook, Instagram, and YouTube	No information available
Diamond-Smith [54]	India	Women aged between 18 and 49 years	Anemia-related knowledge	Facebook	5 months
Chang et al [55]	Zhejiang Province, China	General population	Physical activity and healthy diet	WeChat	No information available

^a^MSM: men who have sex with men.

^b^STI: sexually transmitted infection.

^c^HPV: human papillomavirus.

**Table 2:** Methodologic quality, cost, and funding of included studies.

Study	Social media’s role in this intervention or outcome clearly reported	This role quantified^a^	Social media described as effective	Theoretical model application	Conceptual model application	Cost of intervention reported	Funding source reported	Sources of bias reported
Garett et al [23]	Yes	No	Yes	No	No	No	Yes	Recall
Young et al [24]	Yes	Yes	Yes	No	No	No	Yes	Self-reported
Harding et al [25]	Yes	Yes	Yes	No	No	No	Yes	Nonrepresentative of general population owing to internet or social media access
Cao et al [26]	Yes	No	Yes	No	No	No	No	None
Sap et al [27]	Yes	No	Yes	No	No	No	No	Nonrepresentative of general population owing to internet or social media access; self-reported
Cole et al [28]	Yes	Yes	Yes	No	No	No	Yes	None
Hutchinson et al [29]	No	No	Yes	Transtheoretical model	No	No	Yes	Nonrepresentative of general population owing to internet or social media access
Goldenhersch et al [30]	Yes	Yes	Yes	Contemplation ladder	No	No	Yes	Short follow-up; self-reported
Cool et al, 2015 [31]	Yes	Yes	Yes	No	No	No	Yes	None
Hamill et al, 2015 [32]	Yes	Yes	Yes	No	No	Yes	Yes	None
Jiebing et al [33]	Yes	No	Yes	No	No	No	Yes	None
Mo et al [34]	Yes	No	Yes	Theory of planned behavior	No	No	Yes	Self-reported
Wu et al [35]	No	No	No	No	No	No	Yes	Nonrepresentative of general population owing to internet or social media access
Ahmad et al [36]	Yes	Yes	Yes	Social cognitive theory	Yes	No	Yes	Nonrepresentative of general population owing to internet or social media access; other selection bias
Cavalcanti et al [37]	Yes	Yes	Yes	No	No	No	No	Research team was not blinded to randomization
Chen et al [38]	No	No	No	COM-B^b^ and Behavior Change Wheel framework	Yes	No	Yes	Participants and researchers were not blinded
Todorovic et al [39]	Yes	No	Yes	No	No	No	Yes	No randomization
Chai et al [40]	No	No	No	No	Yes	No	Yes	Lost to follow-up
Lwin et al [41]	No	No	No	Protection motivation theory	Yes	No	Yes	Nonrepresentative of general population owing to internet or social media access
Pereira et al [42]	Yes	Yes	No	No	No	No	No	Nonrepresentative of general population owing to internet or social media access
Gamboa et al [43]	Yes	Yes	Yes	Social cognitive theory	No	No	Yes	Self-reported
Januraga et al [44]	Yes	No	No	Technology acceptance model	No	No	Yes	Nonrepresentative of general population owing to internet or social media access
Thammasarn and Banchonhattakit [45]	No	No	No	No	No	No	Yes	None
He et al [46]	Yes	No	Yes	No	No	No	Yes	None
Souza et al [47]	No	No	No	No	No	No	No	None
Chiu et al [48]	Yes	No	Yes	No	No	No	No	Self-reported; lost to follow-up; nonrepresentative of general population owing to internet or social media access
Chiu et al [49]	Yes	No	No	No	No	No	No	Self-reported; lost to follow-up; nonrepresentative of general population owing to internet or social media access
Purdy [50]	Yes	Yes	Yes	No	No	No	Yes	None
Parsapure et al [51]	No	No	Yes	No	No	No	Yes	None
Wu et al [52]	Yes	No	Yes	No	No	No	Yes	Lost to follow-up
Hutchinson et al [53]	No	Yes	No	No	Yes	No	Yes	Self-reported; interviewer bias
Diamond-Smith [54]	Yes	Yes	Yes	No	No	No	Yes	Selection bias
Chang et al [55]	Yes	Yes	No	No	No	No	No	None

^a^Quantified through clicks, shares, comments, or other method of engagement.

^b^COM-B: Capability Opportunity Motivation Behavior.

### Geographic and Methodological Characteristics of Included Studies

The studies included for review were conducted in geographically diverse LMICs, with China (8/33, 24%), Peru (4/33, 12%, all from the same program), and Brazil (3/33, 9%) being the sites with the most social media–based interventions (Table 3). Other countries included Argentina, Cameroon, the Dominican Republic, Egypt, Ghana, India, Indonesia, Iran, Kenya, Malaysia, Serbia, Sri Lanka, Thailand, the Philippines, and Turkey (Table 1). Of these, 33% (11/33) are upper-middle–income countries and 21% (7/33) are lower-middle–income countries.

Of the 33 studies included in the review, 23 (70%) were limited to a particular subnational locality, typically either a large city or a particular region, and 10 (30%) were designed to be national in scope.

Common study designs included randomized trials (12/23, 52%) and observational studies (11/23, 48%). Only 13% (3/23) of studies were qualitative, and none used mixed methods.

Common focus populations included a particular age group of interest (11/23, 49%), the general population (8/23, 35%), and men who have sex with men (6/23, 26%). The desired behavior change component varied widely, but studies most frequently aimed to change HIV testing and knowledge (5/23, 22%), increase physical activity and weight loss (5/23, 22%), and smoking cessation (5/23, 22%).

Studies have frequently combined types of evaluations, with 6% (2/33) of studies including components related to formative evaluations, 52% (17/33) with process evaluation components, 76% (25/33) with outcome indicators, and 18% (6/33) with impact measurements. In all, 9% (3/33) of studies were described with insufficient detail to clarify the specific type of evaluation conducted.

Although most studies described the overall interventions thoroughly enough that they could be understood (24/33, 73%), few reported social media use with sufficient clarity (19/33, 58%). In 42% (14/33) of studies, the role of social media in greater intervention was quantified using some measure of engagement, including clicks, likes, comments, shares, and retweets. However, the authors concluded that the use of social media was effective (22/33, 67%). Studies, especially those with data on outcomes or impacts, frequently paired data on social media use with a measurement of effectiveness captured outside of social media use, such as changes in anthropometric measurements, knowledge of a specific health topic, or smoking status (22/33, 67%).

**Table 3:** Characteristics of included studies (N=33).

Study characteristics			Studies, n (%)
**Country**			
	China	8 (24)	
	Peru	4 (12)	
	Brazil	3 (9)	
	Other^a^	19 (58)	
**Social media platform^b^**			
	Facebook	17 (52)	
	WeChat	8 (24)	
	Instagram	4 (12)	
	Platform created for intervention	4 (12)	
	YouTube	3 (9)	
	WhatsApp	3 (9)	
	Twitter	3 (9)	
	Reddit	1 (3)	
	Multiple platforms	6 (18)	
**Methodologic quality**			
	The role of social media in this intervention or outcome was clearly reported	24 (73)	
	This role was quantified through clicks, shares, comments, or other method of engagement	14 (42)	
	Social media was described as effective	22 (67)	
	A theoretical or conceptual model was used	10 (30)	

^a^Argentina, Cameroon, the Dominican Republic, Egypt, Ghana, India, Indonesia, Iran, Kenya, Malaysia, Serbia, Sri Lanka, Thailand, the Philippines, and Turkey.

^b^Categories are not mutually exclusive.

Facebook was the most common social media platform used in the studies (17/33, 52%), followed by WeChat (8/33, 24%), Instagram (4/33, 12%), and WhatsApp (3/33, 9%; Table 3). A few studies (4/33, 12%) designed their own apps for use on mobile phones with built-in social media components and 18% (6/33) of studies spread their efforts across several platforms (eg, Facebook, Twitter, and Instagram).

Few studies included a description of either a theoretical model (8/33, 24%) or developed a conceptual model (5/33, 15%) that guided researchers’ efforts in the design or evaluation stages. Although clear information on the cost of the interventions was rare (1/33, 3%), most (24/33, 73%) studies included information on sources of funding.

Although every study focused on an intervention that, by its nature, required access to technology and the internet, relatively few highlighted this as a potential source of selection bias that might lessen an intervention’s external validity (11/33, 33%). This suggests that researchers do not perceive technology access as an obstacle to the effective implementation of such interventions in LMICs.

The length of observation after the intervention ended for most of the studies was relatively short, with no studies following up with their participants for more than a year, and half of the studies (17/33, 52%) did not report any follow-up data.

Finally, none of the studies included in this systematic review reported on the methods used to diminish the possibility of interference or data theft on behalf of the participants from internet service providers, software developers, social media services (where applicable), or other interactive users, despite the sharing of data with a third-party service being a requisite of participation eligibility.

## Discussion

### Principal Findings

In this systematic review of studies on interventions that use social media to encourage health behavior change in LMICs, we evaluated 33 studies across a range of interventions, settings, and techniques. We identified important gaps in the types and sectors of interventions, length of follow-up, evaluation techniques, use of theoretical and conceptual models, and discussions of the privacy implications of social media use. In addition, we found that although social media interventions have been conducted in a number of LMICs worldwide, few have been conducted in the poorest countries and few have been done in sub-Saharan Africa.

The range of interventions described in the studies included in this review was limited. We found that the body of literature on behavior change in LMICs is not able to address the question of whether social media is generally useful in these settings or even appropriate for certain types of behavior change work specifically. These determinations will likely need to be extremely context-specific, given the variation in social media access and willingness to use it for this type of work.

A conspicuous limitation of most of the studies included in this review was the lack of data on long-term outcomes and impact. Behavior change can take time, and the potential for regression to earlier states is well known. Future research should include a longitudinal follow-up to assess the long-term effects of social media behavioral interventions. In addition, there was a lack of evidence on the effectiveness of theories of change in social media interventions, and future research should focus on testing processes of change.

Given the relative novelty of behavior change interventions conducted via social media, the lack of formative research evaluating the feasibility, appropriateness, and acceptability of specific types of projects is troubling. The earliest study to use formative research was published in 2015, many years after social media became widespread. Formative research helps ensure that a specific intervention is likely to be needed, understood, and accepted by the population of interest. Without this critical step, it is possible to miss important modifications that would have helped shape extant interventions into successful social media–based projects [56]. There are examples of such formative studies in high-income countries, but we found no such formative research in this review. A more rigorous application of the principles of program evaluation will help develop targeted, effective social media–based interventions.

One of the strengths of social media interventions is the availability of objective dosage and exposure data from analytics. However, our review found that some studies reported that their social media efforts were effective without clearly reporting quantitative data (eg, clicks, shares, and views) on social media use. Future research should examine the characteristics of engagement exposure to evaluate dose–response effects, that is, to determine whether more exposure or exposure of a specific type is associated with successful behavior change. It is important to objectively attribute intervention effects to observed behavior changes and build an evidence base in the field.

Our review identified important gaps in the application of theoretical and conceptual models to explain why a particular intervention was needed, how it might have been expected to work, and how it could have been evaluated. The relatively low number of studies embracing this method is concerning, especially given the novelty of social media–based interventions. The use of evidence-based behavioral theory and a program-specific model are hallmarks of well-designed interventions, and these methods need to be explicitly included to support evaluations of future social media interventions in LMICs [57].

Finally, this review raises the question of the ethical implications of using a third-party social media service as a medium for conducting public health experiments. Of notable concern are the various ways in which a study participant’s privacy could be violated. When a user interacts with a wide-reaching social media platform (eg, Facebook or Twitter), there are multiple stakeholders who may have a vested interest in harvesting any data or communication provided by the participant: internet service providers, social media platforms, other interactive users, or even a governmental entity with a backdoor encryption policy. Ensuring participant security and protection of privacy are among the most critical components of ethical research; explicit explanation of these risks to personal data loss is necessary to incorporate in every public health social media study.

### Limitations

Although this study was conducted following the PRISMA guidelines, there are some important limitations to this systematic review. We did not conduct any quantitative review of the studies or meta-analysis; therefore, we were unable to provide a quantitative assessment of the effectiveness of the interventions of interest. We also limited our search by not availing ourselves of unpublished literature or literature published in a language other than English, where it is possible that we would have found additional intervention descriptions.

### Conclusions

In conclusion, as social media becomes a more powerful and omnipresent factor in people’s lives, its potential as a platform for public health work has grown rapidly. This systematic review of social media–based behavior change interventions conducted in LMICs highlights the need for diversity and methodological rigor at every step in the planning, implementation, and evaluation stages of programming.

## Appendix

Multimedia Appendix 1 Search string for Embase.

## Abbreviations

LMIC: low- and middle-income country
PRISMA: Preferred Reporting Items for Systematic Reviews and Meta-Analyses
