# Supplementary material for: Social Media–Based Interventions for Health Behavior Change in Low- and Middle-Income Countries: Systematic Review
Source: J Med Internet Res. 2022 Apr 14;24(4):e31889. doi: 10.2196/31889 (PMC9052020; doi:10.2196/31889)
Supplement: Multimedia Appendix 1 [file jmir_v24i4e31889_app1.docx]

**Supplementary materials**

Search string for Embase

(('social media'/exp OR 'twitter'/exp OR 'blogging'/exp OR 'youtube'/exp OR 'facebook'/exp OR 'instagram'/exp OR 'smartphone'/exp OR 'website'/exp OR 'wechat'/exp OR 'whatsapp'/exp OR 'online social network'/exp OR 'digital media'/exp) OR ('social media' OR 'digital platform*' OR 'digital media' OR facebook OR twitter OR youtube OR pinterest OR myspace OR aol OR trillian OR icq OR irc OR wechat OR snapchat OR reddit OR digg OR fark OR linkedin OR discord OR whatsapp OR instagram OR (“social network*” NEAR/2 (site* OR website* OR online))):ti,ab,kw)

**AND**

(('health behavior'/exp OR 'behavior change'/exp OR 'behavior changes'/exp OR 'habit'/exp OR 'goal setting'/exp OR 'health promotion'/exp OR 'lifestyle modification'/exp) OR ((chang* OR modif* OR improv* OR manag* OR interven* OR influenc*  OR adapta* OR health OR seeking) NEAR/3 (behavior* OR behaviour*)) OR ((lifestyle OR life-style) NEAR/2 (change* OR modification*)):ti,ab,kw)

**AND**

(("deprived countries":de,ti,ab OR "deprived country":de,ti,ab OR "deprived nation":de,ti,ab OR "deprived nations":de,ti,ab OR "deprived population":de,ti,ab OR "deprived populations":de,ti,ab OR "deprived world":de,ti,ab OR "developing countries":de,ti,ab OR "developing country":de,ti,ab OR "developing economies":de,ti,ab OR "developing economy":de,ti,ab OR "developing nation":de,ti,ab OR "developing nations":de,ti,ab OR "developing population":de,ti,ab OR "developing populations":de,ti,ab OR "developing world":de,ti,ab OR "lami countries":de,ti,ab OR "lami country":de,ti,ab OR "less developed countries":de,ti,ab OR "less developed country":de,ti,ab OR "less developed economies":de,ti,ab OR "less developed economy":de,ti,ab OR "less developed nation":de,ti,ab OR "less developed nations":de,ti,ab OR "less developed population":de,ti,ab OR "less developed populations":de,ti,ab OR "less developed world":de,ti,ab OR "lesser developed countries":de,ti,ab OR "lesser developed country":de,ti,ab OR "lesser developed economies":de,ti,ab OR "lesser developed economy":de,ti,ab OR "lesser developed nation":de,ti,ab OR "lesser developed nations":de,ti,ab OR "lesser developed population":de,ti,ab OR "lesser developed populations":de,ti,ab OR "lesser developed world":de,ti,ab OR "LMIC":de,ti,ab OR "LMICS":de,ti,ab OR "low gdp":de,ti,ab OR "low gnp":de,ti,ab OR "low gross domestic":de,ti,ab OR "low gross national":de,ti,ab OR "low income countries":de,ti,ab OR "low income country":de,ti,ab OR "low income economies":de,ti,ab OR "low income economy":de,ti,ab OR "low income nation":de,ti,ab OR "low income nations":de,ti,ab OR "low income population":de,ti,ab OR "low income populations":de,ti,ab OR "lower gdp":de,ti,ab OR "lower gnp":de,ti,ab OR "lower gross domestic":de,ti,ab OR "lower gross national":de,ti,ab OR "lower income countries":de,ti,ab OR "lower income country":de,ti,ab OR "lower income economies":de,ti,ab OR "lower income economy":de,ti,ab OR "lower income nation":de,ti,ab OR "lower income nations":de,ti,ab OR "lower income population":de,ti,ab OR "lower income populations":de,ti,ab OR "middle income countries":de,ti,ab OR "middle income country":de,ti,ab OR "middle income economies":de,ti,ab OR "middle income economy":de,ti,ab OR "middle income nation":de,ti,ab OR "middle income nations":de,ti,ab OR "middle income population":de,ti,ab OR "middle income populations":de,ti,ab OR "poor countries":de,ti,ab OR "poor country":de,ti,ab OR "Poor Economies":de,ti,ab OR "Poor Economy":de,ti,ab OR "poor nation":de,ti,ab OR "poor nations":de,ti,ab OR "poor population":de,ti,ab OR "poor populations":de,ti,ab OR "poor world":de,ti,ab OR "poorer countries":de,ti,ab OR "poorer country":de,ti,ab OR "Poorer Economies":de,ti,ab OR "Poorer Economy":de,ti,ab OR "poorer nation":de,ti,ab OR "poorer nations":de,ti,ab OR "poorer population":de,ti,ab OR "poorer populations":de,ti,ab OR "poorer world":de,ti,ab OR "third world":de,ti,ab OR "transitional countries":de,ti,ab OR "transitional country":de,ti,ab OR "Transitional Economies":de,ti,ab OR "Transitional Economy":de,ti,ab OR "under developed countries":de,ti,ab OR "under developed country":de,ti,ab OR "under developed economies":de,ti,ab OR "under developed economy":de,ti,ab OR "under developed nation":de,ti,ab OR "under developed nations":de,ti,ab OR "under developed population":de,ti,ab OR "under developed populations":de,ti,ab OR "under developed world":de,ti,ab OR "under served countries":de,ti,ab OR "under served country":de,ti,ab OR "under served nation":de,ti,ab OR "under served nations":de,ti,ab OR "under served population":de,ti,ab OR "under served populations":de,ti,ab OR "under served world":de,ti,ab OR "underdeveloped countries":de,ti,ab OR "underdeveloped country":de,ti,ab OR "underdeveloped economies":de,ti,ab OR "underdeveloped economy":de,ti,ab OR "underdeveloped nation":de,ti,ab OR "underdeveloped nations":de,ti,ab OR "underdeveloped population":de,ti,ab OR "underdeveloped populations":de,ti,ab OR "underdeveloped world":de,ti,ab OR "underserved countries":de,ti,ab OR "underserved country":de,ti,ab OR "underserved nation":de,ti,ab OR "underserved nations":de,ti,ab OR "underserved population":de,ti,ab OR "underserved populations":de,ti,ab OR "underserved world":de,ti,ab)

OR

(Afghanistan:de,ti,ab OR Albania:de,ti,ab OR Algeria:de,ti,ab OR “American Samoa”:de,ti,ab OR Angola:de,ti,ab OR Argentina:de,ti,ab OR “Argentine Republic”:de,ti,ab OR Armenia:de,ti,ab OR Azerbaijan:de,ti,ab OR Bangladesh:de,ti,ab OR Belarus:de,ti,ab OR Byelarus:de,ti,ab OR Belorussia:de,ti,ab OR Belize:de,ti,ab OR Benin:de,ti,ab OR Bhutan:de,ti,ab OR Bolivia:de,ti,ab OR Bosnia:de,ti,ab OR Botswana:de,ti,ab OR Brazil:de,ti,ab OR Bulgaria:de,ti,ab OR Burma:de,ti,ab OR “Burkina Faso”:de,ti,ab OR Burundi:de,ti,ab OR “Cabo Verde”:de,ti,ab OR “Cape verde”:de,ti,ab OR Cambodia:de,ti,ab OR Cameroon:de,ti,ab OR “Central African Republic”:de,ti,ab OR Chad:de,ti,ab OR China:de,ti,ab OR Colombia:de,ti,ab OR Comoros:de,ti,ab OR Comores:de,ti,ab OR Comoro:de,ti,ab OR Congo:de,ti,ab OR “Costa Rica”:de,ti,ab OR “Cote d Ivoire”:de,ti,ab OR Cuba:de,ti,ab OR Djibouti:de,ti,ab OR Dominica:de,ti,ab OR “Dominican Republic”:de,ti,ab OR Ecuador:de,ti,ab OR Egypt:de,ti,ab OR “El Salvador”:de,ti,ab OR Eritrea:de,ti,ab OR Eswatini:de,ti,ab OR Ethiopia:de,ti,ab OR Fiji:de,ti,ab OR Gabon:de,ti,ab OR Gambia:de,ti,ab OR Gaza:de,ti,ab OR “Georgia Republic”:de,ti,ab OR Georgian:de,ti,ab OR Ghana:de,ti,ab OR Grenada:de,ti,ab OR Grenadines:de,ti,ab OR Guatemala:de,ti,ab OR Guinea:de,ti,ab OR “Guinea Bissau”:de,ti,ab OR Guyana:de,ti,ab OR Haiti:de,ti,ab OR Herzegovina:de,ti,ab OR Hercegovina:de,ti,ab OR Honduras:de,ti,ab OR India:de,ti,ab OR Indonesia:de,ti,ab OR Iran:de,ti,ab OR Iraq:de,ti,ab OR Jamaica:de,ti,ab OR Jordan:de,ti,ab OR Kazakhstan:de,ti,ab OR Kenya:de,ti,ab OR Kiribati:de,ti,ab OR Korea:de,ti,ab OR Kosovo:de,ti,ab OR Kyrgyz:de,ti,ab OR Kirghizia:de,ti,ab OR Kirghiz:de,ti,ab OR Kirgizstan:de,ti,ab OR Kyrgyzstan:de,ti,ab OR “Lao PDR”:de,ti,ab OR Laos:de,ti,ab OR Lebanon:de,ti,ab OR Lesotho:de,ti,ab OR Liberia:de,ti,ab OR Libya:de,ti,ab OR Macedonia:de,ti,ab OR Madagascar:de,ti,ab OR Malawi:de,ti,ab OR Malay:de,ti,ab OR Malaya:de,ti,ab OR Malaysia:de,ti,ab OR Maldives:de,ti,ab OR Mali:de,ti,ab OR “Marshall Islands”:de,ti,ab OR Mauritania:de,ti,ab OR Mauritius:de,ti,ab OR Mexico:de,ti,ab OR Micronesia:de,ti,ab OR Moldova:de,ti,ab OR Mongolia:de,ti,ab OR Montenegro:de,ti,ab OR Morocco:de,ti,ab OR Mozambique:de,ti,ab OR Myanmar:de,ti,ab OR Namibia:de,ti,ab OR Nauru:de,ti,ab OR Nepal:de,ti,ab OR Nicaragua:de,ti,ab OR Niger:de,ti,ab OR Nigeria:de,ti,ab OR Pakistan:de,ti,ab OR Palau:de,ti,ab OR Panama:de,ti,ab OR “Papua New Guinea”:de,ti,ab OR Paraguay:de,ti,ab OR Peru:de,ti,ab OR Philippines:de,ti,ab OR Phillippines:de,ti,ab OR Philipines:de,ti,ab OR Phillipines:de,ti,ab OR Principe:de,ti,ab OR Romania:de,ti,ab OR Rwanda:de,ti,ab OR Ruanda:de,ti,ab OR Samoa:de,ti,ab OR “Sao Tome”:de,ti,ab OR Senegal:de,ti,ab OR Serbia:de,ti,ab OR “Sierra Leone”:de,ti,ab OR “Solomon Islands”:de,ti,ab OR Somalia:de,ti,ab OR “South Africa”:de,ti,ab OR “South Sudan”:de,ti,ab OR “Sri Lanka”:de,ti,ab OR “St Lucia”:de,ti,ab OR “St Vincent”:de,ti,ab OR Sudan:de,ti,ab OR Surinam:de,ti,ab OR Suriname:de,ti,ab OR Swaziland:de,ti,ab OR Syria:de,ti,ab OR “Syrian Arab Republic”:de,ti,ab OR Tajikistan:de,ti,ab OR Tadzhikistan:de,ti,ab OR Tadjikistan:de,ti,ab OR Tadzhik:de,ti,ab OR Tanzania:de,ti,ab OR Thailand:de,ti,ab OR Timor:de,ti,ab OR Togo:de,ti,ab OR Tonga:de,ti,ab OR Tunisia:de,ti,ab OR Turkey:de,ti,ab OR Turkmen:de,ti,ab OR Turkmenistan:de,ti,ab OR Tuvalu:de,ti,ab OR Uganda:de,ti,ab OR Ukraine:de,ti,ab OR Uzbek:de,ti,ab OR Uzbekistan:de,ti,ab OR Vanuatu:de,ti,ab OR Venezuela:de,ti,ab OR Vietnam:de,ti,ab OR “West Bank”:de,ti,ab OR Yemen:de,ti,ab OR Zambia:de,ti,ab OR Zimbabwe:de,ti,ab))
